# Exploring the impact of active learning strategies on learning outcomes and educational experiences in undergraduate nursing education: a qualitative descriptive study

**DOI:** 10.1186/s12909-026-09512-0

**Published:** 2026-05-23

**Authors:** B. Najdi, A. Al-Jawabreh

**Affiliations:** Faculty of Graduate Studies, Arab American University-Ramallah, Ramallah, Palestine

**Keywords:** Active learning, Nursing education, Undergraduate nursing students, Qualitative research, Teaching strategies

## Abstract

**Background:**

Active learning is increasingly promoted in nursing education as a student-centred and andragogical approach that enhances engagement, competence, and clinical reasoning. However, limited qualitative evidence explores how students and educators experience active learning and its influence on learning outcomes within real educational contexts.

**Objective:**

To provide a comprehensive description of how active learning strategies influence learning outcomes and educational experiences among undergraduate nursing students and educators, and to identify multilevel factors shaping their implementation.

**Methods:**

A qualitative descriptive design was employed. Four focus groups were conducted with purposively selected undergraduate nursing students and educators from the Faculty of Nursing at the Arab American University–Palestine (*n* = 17). Interviews were conducted in Arabic, audio-recorded, transcribed verbatim, and translated into English. Data were analysed using Braun and Clarke’s reflexive thematic analysis with NVivo to identify patterns across participants’ perspectives. Trustworthiness was ensured through reflexivity, reflective journaling, and member checking.

**Results:**

Five themes emerged. Participants described exposure to diverse active learning strategies, including team-based learning, simulation, case-based learning, flipped classroom activities, peer learning, and gamification. Active learning was perceived to enhance confidence, engagement, knowledge retention, and clinical reasoning. However, its effectiveness was shaped by multilevel conditions, including learner readiness, psychological safety, instructional facilitation, classroom dynamics, and institutional support. Educators further conceptualized active learning as an andragogical process involving intentional instructional design, experiential learning, and reflective feedback practices.

**Conclusion:**

Active learning was experienced as a meaningful approach that supports competence, engagement, and professional preparedness. The findings highlight the importance of psychologically safe learning environments, structured facilitation, and institutional readiness in shaping the implementation and sustainability of active learning in undergraduate nursing education.

**Supplementary Information:**

The online version contains supplementary material available at 10.1186/s12909-026-09512-0.

## Introduction

Active learning is a teaching approach in which the educator acts as a facilitator, engaging students in activities and discussions to help them retain information and apply it in clinical settings [1]. According to [2], adult learners are self-directed learners who learn most effectively when learning is relevant to their lives, connected to their experiences, and aimed at solving real-life problems, supporting the adoption of andragogical active learning approaches.

The State of the World's Nursing 2020 report emphasizes the need to improve nursing education globally, especially among countries with low or middle incomes where traditional teaching methods remain prevalent, Graduates' willingness to deliver safe and efficient patient care is directly affected by the quality of nursing education [3].

Nursing colleges significantly favored engaging, student-centered approaches, as evidenced by a cross-national survey collected across 17 Middle Eastern countries which found that the most frequently employed methods for active learning were class discussions (89.7%), collaborative learning, and problem-based learning(PBL) (55.1%) [4].

Moreover, Park & Suh (2021) showed in their meta-analysis that examined eighteen studies in nursing education, that active learning approach known as "flipped learning is effective in enhancing learning outcomes for critical thinking (Hedges’ g = 0.87), learning satisfaction (g = 0.79), clinical competence (g = 0.53), and self-directed learning (g = 0.37) [5].

A recent study which was carried out in Oman showed that factors like lack of time, a heavy workload for faculty, lack of training, lack of institutional resources, and student resistance to non-traditional approaches restrict active learning from being employed in nursing education [6].

Successful implementation of active learning in nursing education depends on several key conditions identified in recent literature. Structured instructional support, such as scaffolding, has been shown to enhance student engagement by breaking learning tasks into manageable steps aligned with students’ abilities [7]. In addition, psychological safety plays a critical role, as students are more likely to participate, ask questions, and learn effectively in environments where they feel supported and free from fear of embarrassment [8]. Furthermore, successful implementation depends on broader institutional readiness, including resources, operational support, and appropriate training systems, particularly in resource-constrained and culturally diverse educational context [9].

This study aimed to provide a comprehensive description of how active learning strategies influence learning outcomes and educational experiences among undergraduate nursing students and educators, and to identify multilevel factors shaping their implementation.

This study was guided by the following research questions:

- RQ1. What types of active learning experiences have undergraduate nursing students participated in during their nursing courses?
- RQ2. How do undergraduate nursing students and nurse educators perceive the influence of active learning strategies on students’ learning outcomes?
- RQ3. How do nursing students and nurse educators perceive the facilitators, barriers, and contextual factors influencing the implementation of active learning strategies in nursing education?
- RQ4. How do nurse educators conceptualize active learning and describe its pedagogical purpose in undergraduate nursing education?
- RQ5. What active learning strategies do nurse educators use across theoretical, clinical, and simulation-based teaching, including debriefing and assessment practices, and how do they integrate theory with practice?

## Methodology

A qualitative descriptive design was adopted to explore participants’ experiences with active learning. This approach is particularly suitable for providing a clear and comprehensive account of participants’ perspectives and for examining patterns across different groups. It allows for a rich, practice-oriented understanding of educational experiences and supports the identification of factors influencing implementation.

### Participants and recruitment

Purposive sampling with maximum variation was used to recruit participants who could provide rich and diverse perspectives. The sample included undergraduate nursing students across three academic years and nursing educators from the Faculty of Nursing at the Arab American University–Palestine.

Four focus groups were selected, comprising a total of 17 participants: first-year students (*n* = 4), second-year students (*n* = 4), third-year students (*n* = 5), and educators (*n* = 4). Participants were selected to ensure maximum variation across gender, academic performance, and sociocultural backgrounds for students, and across academic qualifications (MS and PhD), instructional roles (theoretical, clinical, and simulation), and managerial responsibilities for educators.

### Data collection methods

Two semi-structured focus group interview guides were developed, one for undergraduate nursing students and one for nurse educators. The guides were adapted from previous qualitative studies [10, 11] and contextualized to the Palestinian nursing education setting. They were tailored to the study objectives and designed to explore participants’ perspectives, perceptions, learning outcomes, facilitators, and contextual factors influencing the implementation of active learning strategies.

To ensure content validity and clarity, the interview guides were reviewed and validated by three experts in qualitative research. Minor refinements were made based on their feedback. The full guides are provided in appendices A and B.

Before conducting the interviews, the researcher received thorough training, which included interview and communication skills, as well as effective listening and questioning techniques to avoid leading questions.

Prior to the interviews, all participants received a clear explanation of the study’s purpose and procedures. Written informed consent was obtained for participation and audio recording.

The interviews were conducted in Arabic and were audio-recorded, and detailed field notes were taken to capture non-verbal cues and group dynamics, focus group discussions continued until sufficient depth and breadth of information were obtained to address the study research questions.

Audio recordings were transcribed into written text within one month of the interviews and subsequently permanently deleted. Transcripts contained no names or personal identifiers. All data were securely stored on a password-protected computer accessible only to the principal investigator. Each session lasted approximately 60–90 min and was conducted in a quiet, comfortable, and private meeting room at the university, and no one other than the researchers and participants was present during the data collection sessions to ensure confidentiality and allow participants to speak freely.

Discussions were facilitated by the researcher using semi-structured, open-ended questions. All research questions were comprehensively addressed during the focus group sessions, and no repeat interviews were conducted, as sufficient depth of information was obtained.

To maintain confidentiality, all identifying information was removed, and each participant was assigned a unique code in place of names or personal details. All original materials were stored separately and securely on a password-protected device accessible only to the research team.

### Data analysis

Audio recordings were transcribed verbatim within 24 h of each session. Transcripts were translated from Arabic, including different Palestinian dialects, into English using AI-assisted translation (ChatGPT). The translated transcripts were then carefully reviewed and cross-checked by the research team to ensure linguistic accuracy and preservation of meaning. Any discrepancies were discussed and resolved collaboratively.

Data were analyzed using Braun and Clarke’s six-step reflexive thematic analysis approach, which provides methodological flexibility and is well-suited for identifying patterns across heterogeneous participant groups. This approach facilitated the comparison and synthesis of perspectives from both undergraduate nursing students and educators, enabling a comprehensive understanding of shared and divergent perspectives across multiple levels [12].

Transcripts were repeatedly read to achieve immersion and identify meaningful units of text. Codes were generated inductively and organized into categories, themes, and subthemes reflecting participants’ shared and unique perceptions, experiences, learning outcomes, and the multilevel facilitators, constraints, and contextual factors shaping the implementation of active learning. NVivo software (Release 1.7.2, QSR International) supported data management and organization, while manual interpretation ensured depth and contextual understanding. Data analysis was conducted by the primary researcher, with regular review and discussion of codes and themes with the academic supervisor to ensure clarity, credibility, and reflexivity.

To ensure credibility, member checking was conducted after the initial analysis. Participants were provided with their corresponding quotations, codes, and developed themes, along with a summary of the interpretation. They were invited to review and confirm whether the analysis accurately reflected their perspectives. Feedback from participants confirmed the consistency and accuracy of the findings.

### Trustworthiness

To enhance the trustworthiness of the study, several strategies were employed to minimize potential researcher bias. Reflexivity was maintained throughout the research process to ensure awareness of the researcher’s assumptions and their potential influence on data interpretation. Reflective journaling was used to document personal perspectives and monitor potential biases during data collection and analysis [13]. In addition, attention was given to maintaining transparency and consistency in data analysis, supporting the credibility and dependability of the findings. These strategies helped ensure that the results accurately represent participants’ perspectives rather than the researcher’s interpretations [12].

### Characteristics of the respondents

Thirteen undergraduate nursing students participated in the study. Participant characteristics are presented in Table 1.

**Table 1:** Demographic characteristics of student participants (*n* = 13)

Variable	Category	n
Age (years)	Range (Min–Max)	17–24 (Mean = 20.0)
Gender	Male	5
	Female	8
Academic Level	1 st Year	4
	2nd Year	4
	3rd Year	5
Place of Residence	City	7
	Village	3
	Campus	3
Academic Achievement Level	High	6
	Moderate	7

Four nursing educators participated in the study (three males and one female), with teaching experience ranging from 2 to 19 years. Three participants held PhD degrees, while one held a master’s degree

## Presentation of results

The analysis included both student and educator perspectives, despite differences in data collection methods and interview guides. Data from each group were initially analyzed to identify patterns within each dataset, and were then integrated to develop overarching themes that capture the broader patterns of active learning across participants.

This approach allowed for the synthesis of perspectives based on shared meanings and recurring patterns, while still acknowledging group-specific insights. While the analysis did not aim to directly compare the two groups, attention was given to how perspectives aligned or differed within each theme. In presenting the findings, student and educator perspectives are described either separately or together, depending on the nature of the theme, to reflect both shared patterns and group-specific insights.

Theme 1 explored the types of active learning experiences reported by students (RQ1). Theme 2 examined the perceived influence of active learning strategies on learning outcomes from both students’ and educators’ perspectives (RQ2). Theme 3 identified the multilevel facilitators, barriers, and contextual conditions influencing the implementation of active learning strategies (RQ3). Theme 4 addressed educators’ conceptualizations and pedagogical purposes of active learning (RQ4), while Theme 5 described the instructional processes and implementation practices through which educators operationalized active learning across theoretical, clinical, and simulation-based contexts (RQ5).

### Theme 1: the active learning landscape in nursing education

Students across the three focus groups described exposure to a range of active learning strategies in nursing education. The strategies were presented in descending order of frequency based on participants’ reports.

**Fig. 1:**
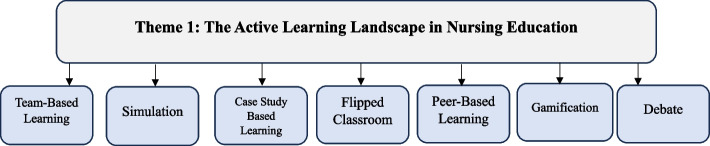
Theme 1: the active learning landscape in nursing education

Figure 1 illustrates the structure of this theme and the active learning strategies identified by students.

Team-based learning was the most commonly mentioned strategy (12 of 50 coded references within the theme). Students highlighted collaboration and shared decision-making, as one participant explained:

“A shared solution. We agree that this is the group’s overall opinion. We write down the main points we will follow, collect the information, present it as a paragraph, and that’s it—we settle on the solution.” (F30)

Simulation was also frequently described and viewed as a modern approach that supports practical learning before clinical exposure. One student noted:

“Active learning, on the other hand, uses more modern approaches, such as the simulation lab in nursing, and practical application before we go down to hospitals.” (F30)

Another participant emphasized the value of hands-on practice, stating: “The closest thing we have at the university is simulation… when I do something with my hands, it is firmly fixed in my mind.” (A20).

Case study–based learning was perceived as highly effective, particularly for remembering information. Students described working in groups to solve cases and present solutions, with one participant stating:

“Yes, like case studies. We have an instructor who does this a lot—he divides us into groups and gives us a case, and we solve it together. Then one of us presents it. It’s impossible to forget the information when we participate this way.” (N99)

The flipped classroom approach was described as involving students in teaching roles, where groups prepare and present content instead of the instructor:

“When I prepare a topic in advance and then explain it during the session, I understand it better and do not forget it… Later, when I encounter a patient with the same condition, I immediately remember what I studied and how to apply it.” (B13)

Peer-based learning was viewed as an instructional attempt to introduce new ways of sharing information among students, as reflected by one participant:

“Meaning from my point of view, the instructor wants to give us new methods in teaching or in giving information to the rest of your colleagues.” (E50)

Less frequently mentioned strategies included gamification and debate, which were still described as engaging. Gamification was associated with competition:

“Kahoot … it is competitive, the one who answers faster gets more points.” (X39)

Debate was valued for encouraging thinking and exposure to different perspectives:

“Because we hear different points of view, and it’s like we are thinking and making connections. It broadens our perspectives and makes us think more.” (E51)

### Theme 2: fostering learning outcomes through active learning

Both students and educators consistently described active learning as a powerful approach that enhances learning outcomes and prepares students for professional nursing practice. The following section first presents students’ perspectives, followed by educators’ perspectives.

Analysis across academic levels showed some variation in the frequency with which learning outcomes were discussed. First-year students most frequently emphasized learning motivation and engagement, whereas second- and third-year students more commonly highlighted enhanced confidence and perceived competence, along with the development of clinical reasoning skills. Knowledge retention was reported across all academic levels, although with varying frequency.

**Fig. 2:**
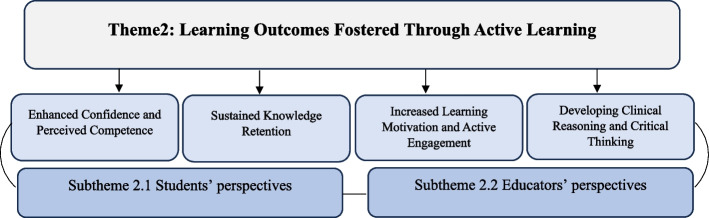
Theme 2: learning outcomes fostered through active learning

Figure 2 illustrates the structure of this theme and the learning outcomes associated with active learning as described by both students and educators.

One important outcome was the development of clinical reasoning and critical thinking. Students explained that active learning pushed them to think more deeply and not just give routine answers. As one student shared:

“I try to gather all the data, and I also exert effort because I have to think a little outside the box—going beyond ordinary thinking—to provide the patient with the best possible outcome.” (A30)

Active learning also helped students feel more confident and competent, especially during simulation activities. Students spoke with strong emotions about these experiences and described feeling capable of treating patients correctly. One participant expressed this clearly:

“I felt like I was flying, like I was in the sky… You feel that you are able to treat the patient correctly… We make mistakes here, apply here, and then go to the hospital to see this in real life, which leads to less harm and more effective treatment for the patient.” (A20)

Another important outcome was increased motivation and active engagement. Students explained that when learning was interactive and enjoyable, they were more focused and eager to participate. Gamified activities, such as Kahoot, an online platform that fosters competition between students (www.kahoot.com) [14], were often mentioned as motivating:

“I really liked the Kahoot game, it increased my concentration and gave me motivation. Every correct answer pushed me to achieve higher levels… and the idea reached me in a very enjoyable way.” (E50)

Students also described how active learning supported long-term knowledge retention. Learning through practice, repetition, and making mistakes helped information stay in their minds. One student explained:

“Making mistakes is not embarrassing; on the contrary, it strengthens learning… those moments stay in memory and help fix the information.” (C60)

Another student highlighted how repeated practice in simulation made learning last beyond the classroom:

“It becomes firmly fixed in my mind. I practiced this idea once, twice, three times in the simulation lab. When I went to apply it in real practice, I was fully competent.” (F30)

Educators similarly emphasized that active learning played a critical role in enhancing students’ confidence and perceived competence. Instructors observed that when students actively participate in preparing, presenting, and applying clinical tasks, they demonstrate stronger performance and increased self-confidence. One educator noted:

“When we ask each student to prepare beforehand and then apply it with their own hands, I find that in the end they perform much better.” (I3)

Educators also described how active learning strategies contribute to sustained knowledge retention by linking theoretical instruction with clinical application. Reflective activities such as debriefing were viewed as particularly important for consolidating learning and transforming clinical experiences into deeper, long-term understanding:

“Debriefing helped students engage in reflective thinking, correct mistakes, consolidate learning and turning the clinical experience into deeper and more sustainable learning.” (I1)

Another frequently discussed outcome was increased learning motivation and engagement. Educators highlighted the value of collaborative case discussions, peer-based learning, and structured group activities in fostering interaction, responsibility, and enthusiasm among students:

“If you start the lecture with a case and present it at the beginning, and you get solutions or different opinions from students, this motivates them to start the lecture. You feel that students become engaged and very enthusiastic.” (I5)

In addition, educators consistently emphasized the role of active learning in developing clinical reasoning and critical thinking. Instructional strategies such as questioning, simulation-based scenarios, and analytical discussion were intentionally used to move students beyond memorization toward deeper reasoning and evidence-based decision-making:

“You are always trying to get the student to think, analyze, and understand the concept, not memorize it.” (I2)

Overall, educators’ accounts closely aligned with students’ perspectives, reinforcing the perceived effectiveness of active learning in strengthening confidence, engagement, knowledge retention, and higher-order clinical reasoning skills.

These findings demonstrate a strong alignment between students’ and educators’ perspectives regarding the learning outcomes associated with active learning approaches.

### Theme 3: multilevel facilitators and constraints shaping active learning experiences

**Fig. 3:**
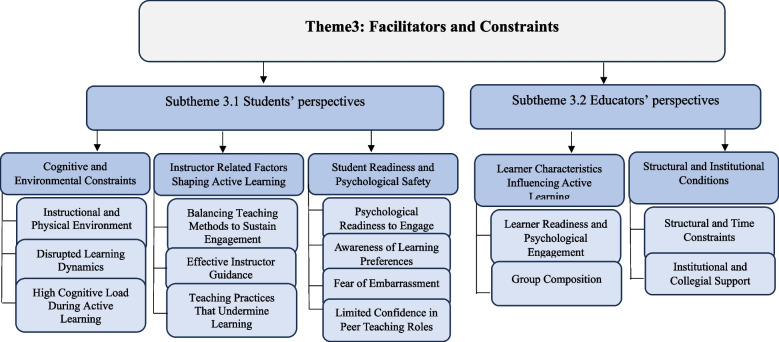
Theme 3: multilevel facilitators and constraints shaping active learning experiences

Both students and educators identified multiple factors that facilitated or constrained engagement in active learning. As illustrated in Fig. 3, these influences operated across several levels, including environmental conditions, instructor-related practices, learner readiness, and institutional factors. Students’ perspectives are presented first, followed by educators’ perspectives.

Variation across academic levels was observed in the factors influencing active learning experiences. First-year students more frequently emphasized instructional and environmental constraints and the need for psychological readiness, reflecting early-stage adjustment to active learning approaches. In contrast, second- and third-year students more often highlighted instructor-related factors, particularly the importance of effective instructional guidance and balanced teaching methods. Concerns related to fear of embarrassment and confidence in peer-learning roles were reported across all academic levels, indicating that psychological safety remains an ongoing factor influencing engagement throughout the undergraduate program.

#### Subtheme 3.1: students’ perspectives

##### Category 3.1.1: cognitive and environmental constraints

Some students described active learning as mentally demanding, especially when they felt unprepared or expected to know everything in advance. One student explained this pressure clearly:

“I felt that I had to be fully knowledgeable about the topic. I wanted to anticipate that a student might ask me something because the doctor had not explained the topic… or the doctor himself might ask me.” (N12)

Environmental and time-related issues also affected students’ ability to engage. Several students described feeling sleepy or bored due to the physical setting or long lectures. One participant shared:

“When they turn off the lights, I get very sleepy… This is not active learning at all… maybe they could take us outside, like on a trip… changing the environment. Not the same routine, the same lecture, the same activities all the time.” (F30)

Others emphasized that long lectures reduced motivation and concentration:

“When the lecture becomes long… students honestly get bored… their learning motivation decreases, and attention and concentration decrease.” (E50)

Instructor energy and movement were also mentioned as important for maintaining focus:

“I like the instructor to stand, move around, and explain with a loud voice.” (E51)

Group discussions, while valuable, were sometimes described as difficult to manage. Some students felt that discussions could turn chaotic or lose seriousness:

“Discussion might create chaos, so the instructor must have control.” (Z40)

“When we are in groups and we are all friends, sometimes the topic loses its seriousness… and this can affect our understanding of the material.” (E51)

##### Category 3.1.2: instructor-related factors shaping active learning

Instructor practices were described as a key factor influencing students’ engagement. Some students felt that nursing education should rely heavily on active learning, given its practical nature:

“No, I feel that everything needs to be active, because nursing is work.” (A30)

Students also highlighted that enjoyment mattered more than grades alone:

“To make fun.” (X39).

“It’s not all about marks… we also want enjoyment, that the student likes the subject, not just pressure of marks.” (R55)

Supportive instructor guidance was highly valued. Students appreciated instructors who corrected mistakes without criticism and focused on improvement:

“He does not criticize anyone. He only corrects us… The goal is clearly to improve the student’s performance.” (I44)

Even when a topic was not interesting, the instructor’s teaching style could make a difference:

“A topic might not be appealing at all, but the instructor’s explanation, teaching style, or the activity they use… can really grab attention.” (C60)

In contrast, some teaching practices were described as discouraging. Students reported boredom when activities were repetitive:

“If we keep doing the same process in the same way, that’s when boredom starts.” (F30)

Others described negative experiences when instructors used pressure or embarrassment:

“I felt the instructor was asking questions to embarrass me, not to help me understand… I didn’t retain the information… I don’t remember the content at all.” (C60)

Time pressure also led some students to focus on finishing tasks rather than learning:

“I just do it to finish it… then I forget it afterward.” (E51)

##### Category 3.1.3: student readiness and psychological safety

Students’ engagement in active learning was strongly influenced by their personal learning preferences and sense of psychological safety. Some students explained that they learned better through practice rather than memorization:

“I am a person who learns what I do, not what I memorize.” (X39)

Visual and interactive elements were also described as helpful:

“When you see pictures, the image… the term stick in your mind.” (E50)

Learning with others sometimes helped students feel closer to the information:

“When we are in a group… the information truly feels closer to me.” (C60)

However, not all students felt comfortable with group work:

“I am a person who does not support and does not like to be in a group honestly.” (X39)

Some students reported that awareness of their learning preferences influenced their engagement in active learning activities. For example, one participant explained that students with auditory learning preferences sometimes found group-based activities distracting, suggesting that instructional strategies should consider variability in learners’ preferences: *“Now it turned out that my style is auditory… I feel active learning sometimes is disturbing… if there are many auditory learners, it is better not to do groups.” (Z40)*

Fear of embarrassment was a common barrier. Students described feeling tense or withdrawn when criticized in front of others:

“When the instructor criticizes me during the presentation, I feel tense the entire time and unable to speak.” (E51)

Others described a sequence of negative feelings that affected learning:

“Confusion first, then embarrassment, then feeling ‘not good enough’… I didn’t feel that the information was retained.” (C60)

Some students avoided asking questions to protect their peers:

“We do not want to embarrass our colleague by saying we did not understand.” (B30)

Confidence in peer teaching was also limited for some students. Several preferred learning directly from instructors:

“I feel more confident when I do it myself.” (A30)

“When the doctor explains, it is better than a colleague presenting.” (B13)

Finally, students described psychological readiness as something that changes over time and depends on mood, experience, and responsibility:

“As a first-year student… I reject group work.” (X39)

“My feeling is not incapacity, but rather a bit of weakness.” (E50)

“My mood is important… it affects me.” (I44)

#### Subtheme 3.2: educators’ perspectives

Educators identified several multilevel factors influencing the effectiveness of active learning implementation, including learner characteristics, group dynamics, and structural and institutional conditions.

##### Category 3.2.1: learner characteristics influencing active learning

Educators emphasized that students’ psychological readiness, confidence, and openness to feedback significantly shaped their engagement in active learning activities. Some students were described as reluctant to participate due to shyness or fear of criticism:

“Some students are shy to stand in front of their peers; some prefer to remain seated.” (I5)

Others were hesitant to join stronger groups because of low self-confidence:

“A weaker student might say, ‘I consider myself the weakest one… I would ruin that group,’ so they join a weaker group to feel at the same level.” (I3)

Educators also observed that readiness to engage varied across academic levels:

“The younger they were, the easier it was for them to accept it… older students sometimes feel embarrassed to make mistakes.” (I3)

Group composition was also described as a critical factor influencing participation and interaction. Educators explained that carefully structured groups, supportive peer relationships, and balanced skill distribution enhanced collaboration and engagement, while poorly composed groups could limit participation and reduce the effectiveness of active learning strategies.

“What makes the biggest difference is the composition of the student group.” (I3)

##### Category 3.2.2: structural and institutional conditions

Educators further identified structural and institutional constraints as key challenges affecting the consistent implementation of active learning. Time limitations, large class sizes, and dense curricular content were frequently mentioned as barriers that restricted the extent to which interactive teaching strategies could be applied:

“The number of students plays a major role… you want to implement active learning, but at the same time you are expected to finish the required content.” (I2)

While large class sizes were often described as a constraint affecting time management and coverage of course content, some educators noted that larger student cohorts could also create opportunities for richer interaction and competitive engagement. As one educator explained:

“Competition is stronger… when it is nursing only, like a full cohort… Competition becomes stronger that way.” (I3)

Despite these constraints, institutional and collegial support were described as important enabling factors. Program coordinators, faculty workshops, and collaborative meetings among instructors were perceived as essential mechanisms that supported the adoption and sustainability of active learning practices within nursing education programs.

“When there is a program coordinator and clear guidance… things become easier and more successful.” (I2)

Together, students’ and educators’ perspectives highlight that the effectiveness of active learning is shaped not only by instructional strategies but also by learner readiness, classroom dynamics, and institutional conditions.

##### Overarching theme: educators’ andragogical conceptions and practices of active learning

**Fig. 4:**
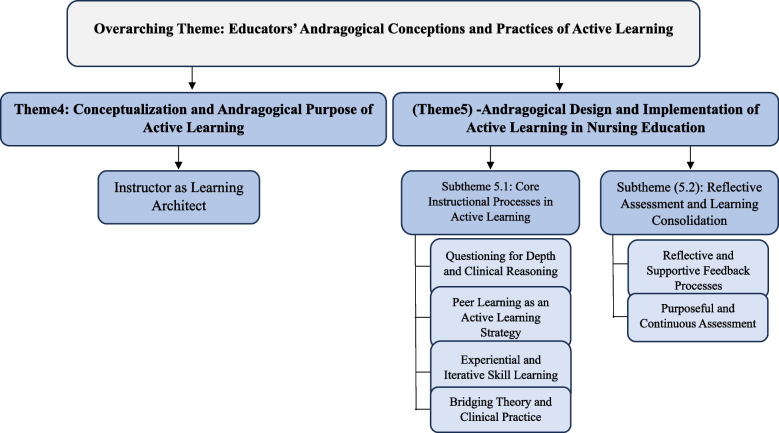
Overarching theme: educators’ andragogical conceptions and practices of active learning

Educators described active learning not only as a set of instructional techniques but as an andragogical philosophy in which the instructor assumes the role of a learning architect who intentionally designs learning environments that promote participation, responsibility, and experiential engagement. As illustrated in Fig. 4, educators consistently emphasized their responsibility for structuring learning experiences, guiding group dynamics, and creating psychologically supportive environments that enable students to actively construct knowledge.

### Theme 4: conceptualization and andragogical purpose of active learning

Educators conceptualized active learning as a learner-centered process that shifts the instructor’s role from information transmitter to facilitator of learning experiences. Participation, preparation, and discussion were viewed as central mechanisms through which students become active partners in the educational process:

“The instructor is supposed to be a facilitator rather than a lecturer… guiding the discussion, asking questions, and encouraging thinking.” (I2)

Several educators described deliberately designing sessions in which students prepare content in advance and participate in discussion-based learning activities:

“I usually tell the students in advance that the next lecture will be a discussion, not a lecture.” (I3)

In this perspective, instructors intentionally shaped classroom structures, group organization, and learning activities to enhance engagement and support learners with varying levels of confidence and readiness. Active learning was also viewed as an essential mechanism for narrowing the gap between theoretical instruction and clinical practice, enabling students to develop professional competence through guided participation and experiential exposure.

### Theme 5: andragogical design and implementation of active learning in nursing education

#### Subtheme 5.1: core instructional processes in active learning

Educators described multiple instructional processes used to operationalize active learning, including questioning strategies, peer-based learning, experiential skill practice, and activities that bridge theory with clinical application.

Questioning techniques were frequently used to stimulate clinical reasoning and analytical thinking:

“I ask them why… think with me.” (I3)

Peer learning was also widely employed to enhance collaboration and responsibility within learning activities:

“Students learn from their peers… distribute roles among themselves and work together.” (I5)

Experiential and iterative skill learning, particularly through simulation and repeated practice, was described as a core instructional component that allows students to learn from mistakes and progressively refine clinical competence. This was reflected in educators’ emphasis on hands-on practice and learning through repetition and error:

“I want the student to do it themselves, even if it doesn’t work the first time. They repeat it, make mistakes, and learn from them. I ask them to apply it and show me how they work with their hands.” (I3)

In addition, educators emphasized the importance of linking theoretical instruction with clinical experiences to ensure continuity between classroom learning and real-world practice. This was clearly reflected in educators’ accounts of integrating theory into clinical application:

“I make the student link theory and clinical practice together. The things they learn in theory, they come and apply them in the clinical setting and explain why.” (I3)

In addition, educators emphasized the importance of coordination between theoretical instructors and clinical trainers to ensure alignment between classroom instruction and clinical practice. Regular preparatory meetings were conducted prior to clinical training periods, during which course learning outcomes (CLOs), instructional priorities, and weekly training objectives were reviewed to ensure that clinical experiences reinforced recently covered theoretical content:

“We bring the trainers together before the training begins… discussing the course content, the CLOs, and the intended learning outcomes.” (I5)

Clinical educators also described the use of structured procedural teaching methods, such as Peyton’s four-step approach, to support progressive skill acquisition. This method allowed students to observe procedures, guide the instructor verbally, and eventually perform the skills independently, thereby strengthening competence and confidence in clinical practice:

“I used Peyton’s method… the student observes, then guides step by step, and finally performs the procedure independently.” (I1)

#### Subtheme 5.2: reflective assessment and learning consolidation

Educators highlighted reflective feedback and formative assessment as essential mechanisms for consolidating learning during active learning activities. Structured debriefing, peer feedback, and reflective discussion were commonly used to support students’ critical reflection and continuous improvement:

“After every procedure… there was structured debriefing about what was done correctly and what could be improved.” (I1)

Continuous formative assessment, including participation-based evaluation, performance-based assessment, and symbolic grading incentives, was also described as a strategy that increased engagement and encouraged consistent preparation for learning activities.

Educators also described the importance of continuous feedback between students and instructors to enhance teaching practices. Several instructors reported actively collecting students’ feedback regarding teaching strategies, session pacing, and learning activities, and using this feedback to refine instructional approaches in subsequent sessions:

“Students write constructive feedback… I read these at home and return to the class the next day to discuss the comments.” (I5)

Overall, these findings provide a comprehensive understanding of both students’ and educators’ perspectives, highlighting how active learning is perceived and interpreted within real educational settings.

## Discussion

This qualitative case study explored the impact of active learning strategies on learning outcomes and educational experiences among undergraduate nursing students and educators. Overall, the findings demonstrate a strong alignment between students’ and educators’ perspectives, indicating that active learning is not only perceived as an effective instructional approach for enhancing engagement, confidence, knowledge retention, and clinical reasoning, but also as an essential andragogical framework shaping modern nursing education. At the same time, the findings highlight that the successful implementation of active learning is influenced by multilevel factors, including learner readiness, instructional design, classroom dynamics, and institutional support structures.

The findings revealed that students were exposed to a broad spectrum of active learning strategies, particularly team-based learning, simulation, and case-based learning, which were identified as the most frequently experienced instructional approaches. In addition, these strategies incorporated various digital and multimedia tools, particularly gamified platforms such as Kahoot [14] and simulation-based technologies, gamified tools were especially effective in enhancing student motivation, engagement, and providing immediate feedback during theoretical sessions. In contrast, simulation-based technologies, particularly high-fidelity simulation, were perceived as more effective in supporting experiential learning, clinical reasoning, and the integration of theory into practice. These findings suggest that the effectiveness of digital tools is contingent upon their alignment with specific learning objectives and structured instructional design.

This is supported by a randomized controlled trial by Othman et al. (2024), which demonstrated that the integration of gamification (e.g., Kahoot) and augmented reality significantly improved nursing students’ knowledge, motivation, and self-efficacy compared to traditional teaching methods [15].

The prominence of these strategies reflects a gradual shift within nursing education toward more interactive, practice-oriented pedagogies that emphasize collaboration, experiential engagement, and applied problem-solving rather than passive knowledge transmission. These findings are consistent with previous research conducted across nursing education contexts, which highlights the increasing adoption of collaborative and experiential learning methods as central components of student-centered curricula [4, 16]. From an andragogical perspective, the widespread presence of these approaches supports the assumptions of adult learning theory [2], which emphasizes the importance of learner participation, experiential learning, and relevance to professional practice. By actively engaging students in authentic clinical scenarios, shared decision-making processes, and applied learning tasks, these strategies appear to facilitate deeper cognitive processing, promote responsibility for learning, and support the development of competencies required for contemporary nursing practice.

The qualitative findings from both students and educators consistently indicate that active learning approaches play a central role in fostering key learning outcomes, including enhanced confidence, perceived competence, sustained knowledge retention, increased engagement, and the development of clinical reasoning and critical thinking skills.

A developmental pattern across academic levels also emerged. Early-stage students appeared to benefit most from structured engagement-focused strategies such as flipped classroom, peer-assisted learning and gamified quiz-based activities which primarily enhanced motivation, engagement, and foundational knowledge acquisition. Studies integrating gamified learning components within flipped-classroom environments report that 78% of students experienced increased learning motivation, alongside significant academic performance improvements [17], reinforcing first-year participants’ narratives describing gamified activities as enjoyable, motivating, and concentration-enhancing learning experiences. Similarly, peer-assisted learning programs implemented with early-level nursing cohorts reported significant gains in engagement and learning confidence, indicating that collaborative and scaffolded learning strategies are particularly effective during the early academic transition period [18]. These findings align with the qualitative accounts of first-year participants in the present study, who emphasized motivation, enjoyment, and active participation as key learning outcomes.

As students progress into higher academic levels, analytical and experiential approaches—such as case-based learning, problem-based learning, and simulation-based clinical scenarios—demonstrated stronger effects on clinical reasoning, competence, and applied decision-making outcomes. A PBL intervention among undergraduate nursing students in Egypt showed very large improvements in knowledge and metacognitive learning outcomes across repeated measurements as indicated by high partial eta-squared (η^2^p ≥ 0.80) [19], while simulation-based education studies reported large-to-very-large improvements in clinical performance (Cohen’s d = 1.74, indicating a very large effect size) [20]. These quantitative outcomes correspond closely with the qualitative findings in the current study, where second-year students highlighted increased competence, confidence, and clinical reasoning skills following active learning exposure.

Studies focusing on senior or clinically advanced students demonstrated that high-fidelity simulation and virtual simulation produced the strongest outcomes related to clinical competence, confidence, and real-practice readiness, with some interventions reporting confidence-related effect sizes exceeding d = 2.00 [21]. Educators in the present qualitative findings similarly emphasized that hands-on application, debriefing, and reflective clinical discussion were particularly effective in preparing advanced students for professional practice. This alignment between learner perspectives, instructor observations, and intervention-based quantitative evidence provides robust triangulated support for the effectiveness of active learning strategies in strengthening both foundational and advanced learning outcomes across undergraduate nursing education.

While the previous findings highlighted the positive learning outcomes mainly associated with active learning approaches [16, 17, 19–21], the present findings demonstrate that the effectiveness of active learning is shaped further by interacting multilevel conditions, including learner readiness, instructor practices, environmental characteristics, and institutional structures. These results are consistent with health-professions education research indicating that active learning outcomes are not determined solely by the instructional strategy itself but also by the psychological, organizational, and contextual environments in which learning occurs [22].

At the learners’ level, both students and educators in the current study emphasized the importance of psychological readiness, confidence, and psychological safety in shaping engagement. Students’ reports of fear of embarrassment, hesitation to participate in peer-teaching roles, and reduced engagement when criticism occurred publicly closely align with previous findings demonstrating that psychologically unsafe classroom climates significantly reduce participation in collaborative and discussion-based learning environments [22]. In addition, variations in students’ learning preferences highlighted the importance of aligning teaching strategies with learners’ preferred learning modalities, as mismatches between instructional methods and learning preferences were sometimes perceived as reducing concentration and engagement, a finding consistent with recent nursing education research emphasizing the role of learning-style–responsive instructional design in enhancing student engagement [23].

Conversely, in this study, both students and educators emphasized that supportive instructor feedback, respectful facilitation, and structured peer collaboration enhanced students’ willingness to participate and engage in reflective dialogue. This finding is consistent with previous qualitative research, which also identified supportive facilitation and structured guidance as key factors promoting student engagement in active learning environments [24].

Environmental and structural constraints identified in this study, including long lecture durations, physical classroom conditions, and time pressure, further mirror previous research highlighting that large class sizes, dense curricula, and limited instructional time frequently restrict instructors’ ability to consistently apply interactive learning strategies [25]. Educators in the current study similarly described the challenge of balancing curriculum coverage with interactive teaching approaches, emphasizing the need for institutional flexibility, coordinated curriculum planning, and collegial support to sustain active learning practices across courses and academic levels.

To further explain how learning can be effectively supported despite these challenges,

Vygotsky’s social constructivist theory highlights that learning happens through interaction with others and is most effective within the Zone of Proximal Development (ZPD), where learners benefit from guidance. This idea forms the basis of scaffolding, which provides structured support to enhance engagement and gradually build independence [26].

Building on this theoretical perspective, these findings further highlight the importance of scaffolding in active learning environments, providing structured guidance and gradual support which may help students to manage cognitive load and to engage more effectively in complex learning tasks. Scaffolding enables learners to progressively build confidence and develop their skills, particularly, when navigating demanding learning contexts or time-constrained instructional settings. This is especially relevant in light of our findings related to students’ readiness and the need for clear instructional support during active learning activities [27].

Instructor-related practices emerged as another critical determinant of engagement. Students emphasized the importance of energetic teaching styles, balanced instructional methods, and supportive correction of mistakes, while educators highlighted the need for structured facilitation and careful group composition. These findings correspond with existing small-group learning research demonstrating that effective facilitation skills, clearly structured activities, and intentional group design significantly enhance participation, collaborative interaction, and learning outcomes [22]. Poorly structured discussions or repetitive activities, as reported by participants in this study, were described as reducing motivation and leading to superficial engagement, reinforcing the importance of instructional design quality in active learning environments. This finding is consistent with previous qualitative research, which also identified insufficiently structured learning activities as a barrier to student engagement in active learning environments [25].

Importantly, variation across academic levels observed in the present study suggests that facilitators and barriers evolve throughout the undergraduate trajectory. First-year students more frequently emphasized environmental comfort, psychological readiness, and adjustment to participatory learning formats, whereas more senior students focused on instructional quality, facilitation effectiveness, and meaningful clinical application. This developmental pattern suggests that early academic stages may require stronger psychological and environmental support structures, while later stages depend more heavily on advanced facilitation strategies and experiential learning integration. This developmental pattern is consistent with previous nursing education research demonstrating that student engagement, learning approaches, and educational needs vary across academic levels, with differences observed between early-year and senior students [28, 29].

Overall, the alignment between the present qualitative findings and prior health-professions education literature reinforces the understanding that successful implementation of active learning depends on a multilevel Learning environment of support, including psychologically safe learning environments, skilled instructional facilitation, well-structured collaborative activities, and enabling institutional conditions. Addressing these interconnected factors is essential for maximizing the educational impact and sustainability of active learning strategies within undergraduate nursing education.

The present findings suggest that educators conceptualize active learning as an andragogical process in which the instructor intentionally designs participatory learning environments that promote learner’s responsibility, experiential engagement, and collaborative knowledge construction. This perspective is congruent with Knowles’ Adult Learning Theory, which emphasizes self-directed learning, experiential learning, readiness to learn, and the application of knowledge to real-life contexts [2]. Recent qualitative research further supports this orientation, demonstrating that structured facilitation, guided participation, and clearly organized experiential activities significantly enhance learner engagement and professional competence development in nursing education [24, 25]. These findings reinforce the view that educators’ instructional design practices—particularly those that promote autonomy, reflection, and experiential participation—represent key mechanisms through which andragogical learning principles are operationalized in contemporary active learning environments.

In this study, findings indicate that the effectiveness of active learning implementation depends not only on the selection of interactive instructional strategies but also on the intentional andragogical design of instructional processes, including questioning techniques, peer-based learning, experiential skill practice, and structured reflective assessment. These instructional processes closely reflect contemporary health-professions education research demonstrating that guided questioning, collaborative learning structures, and experiential practice cycles significantly enhance clinical reasoning, knowledge integration, and professional competence development [30]. In particular, structured procedural teaching models such as Peyton’s four-step approach and iterative simulation-based practice have been shown to support progressive skill acquisition by enabling learners to observe, articulate, and independently perform clinical procedures, thereby strengthening both competence and confidence [31].

Furthermore, our findings highlight the critical role of reflective assessment processes—including structured debriefing, formative feedback, and continuous feedback loops—in consolidating learning and supporting ongoing instructional refinement. Prior open-access education research similarly emphasizes that formative assessment, reflective dialogue, and learner feedback mechanisms function as essential components of effective active learning implementation, enabling both learners and educators to continuously adapt teaching practices and learning strategies to achieve intended educational outcomes [32]. Collectively, these findings suggest that active learning effectiveness is not solely determined by the presence of interactive activities but by the extent to which these activities are embedded within a coherent andragogical design framework that integrates instructional sequencing, experiential learning cycles, and reflective assessment processes.

Collectively, these findings point out that the educational value of active learning lies not only in the use of interactive strategies themselves but in the intentional alignment between learner readiness, facilitation practices, instructional design, and institutional conditions that sustain meaningful engagement. Understanding how these elements interact across different stages of the undergraduate trajectory offers important insight into how active learning can be strategically implemented to support progressive competence development and long-term professional preparedness in nursing education.

## Strengths and limitations

This study has some limitations which did not compromise the overall findings. First, the findings are based on participants’ personal experiences and perceptions, which may differ from actual classroom practices. Second, the study reflects the context of a specific nursing program, and learning environments in other institutions may differ in resources, teaching culture, and exposure to active learning strategies. Finally, the study explored how active learning was experienced rather than how consistently it was implemented across courses, which may influence how students and educators perceived its effectiveness.

## Conclusion

This qualitative case study provides in-depth insight into how active learning is experienced within undergraduate nursing education. The findings suggest that active learning is perceived as a meaningful andragogical approach that supports engagement, knowledge retention, confidence, and clinical reasoning, while also fostering learner participation and experiential learning. Importantly, the findings highlight that the implementation of active learning is shaped by multilevel conditions, including learner readiness, instructional design, facilitation practices, and institutional support. Together, these insights underscore the importance of intentionally designed, psychologically supportive, and experientially oriented learning environments in preparing nursing students for contemporary clinical practice.

## Implications for nursing education

The findings suggest several implications for nursing education practice. First, nursing programs should adopt developmentally sequenced active learning strategies that align instructional approaches with students’ academic progression, emphasizing engagement-oriented methods in early stages and experiential clinical reasoning strategies in later stages. Second, faculty development initiatives should focus on strengthening educators’ facilitation skills, reflective feedback practices, and andragogical instructional design competencies to ensure effective implementation of active learning approaches. Third, institutional support mechanisms, including curriculum coordination, time allocation for interactive teaching, and structured collaboration between theoretical and clinical instructors, are essential for sustaining the effectiveness of active learning initiatives across nursing programs.

**Fig. 5:**
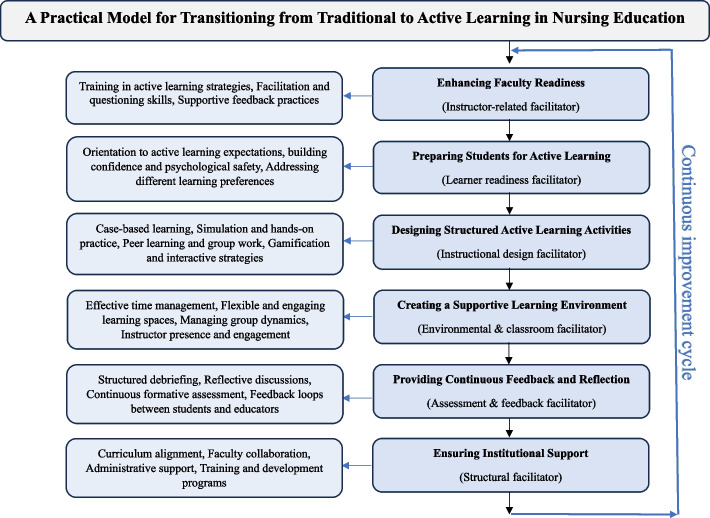
A facilitator-based model for transitioning from traditional to active learning in nursing education

To enhance the practical applicability of these findings, a conceptual model, developed by the authors based on the study findings, is presented to illustrate the transition from traditional to active learning based on the identified facilitators (Fig. 5).

## Supplementary Information

Supplementary Material 1.

## Data Availability

The datasets generated and analyzed during the current study are not publicly available due to participant confidentiality considerations but are available from the corresponding author upon reasonable request. The study instruments are provided in the Appendixes.

## References

[1] Doolittle P, Wojdak K, Walters A. Defining Active Learning: A Restricted Systemic Review. TLI. 2023;11. 10.20343/teachlearninqu.11.25.

[2] Knowels MS. Andragogy in action: applying modern principles of adult education. San Francisco, CA: CA: Jossey-Bass; 1984.

[3] State of the World’s Nursing 2020: Investing in Education, Jobs and Leadership. 1st ed. Geneva: World Health Organization; 2020. 1 p.

[4] AlRuthia Y, Alhawas S, Alodaibi F, Almutairi L, Algasem R, Alrabiah HK, et al. The use of active learning strategies in healthcare colleges in the Middle East. BMC Med Educ. 2019;19(1):143. 10.1186/s12909-019-1580-4. 31088430 10.1186/s12909-019-1580-4 PMC6518770

[5] Park I, Suh Y. Meta-analysis of flipped learning effects in nursing education. Int J Environ Res Public Health. 2021;18(23):12814. 10.3390/ijerph182312814. 34886540 10.3390/ijerph182312814 PMC8657693

[6] Syeda S, D’Costa MP, Swarnadas GS, Leccio BJM, Al-Shizawi WIS, Faraj AM. Active learning strategies: faculty use and their perceived barriers. J Health Allied Sci NU. 2025;15:461–9. 10.25259/JHS-2024-8-28-R1-(1540).

[7] Coffman S, Iommi M, Morrow K. Scaffolding as active learning in nursing education. Teach Learn Nurs. 2023;18(1):232–7. 10.1016/j.teln.2022.09.012. 36339966 10.1016/j.teln.2022.09.012 PMC9627530

[8] Kajan K, Abbasi N, Loizou C. Bridging the silence: understanding motivations and participation barriers in transnational engineering education. Educ Sci Basel. 2025;15(9):1185. 10.3390/educsci15091185.

[9] Khraisat F, Khrais GM. Simulation-based learning in nursing education: current evidence and future directions. Inquisiva Open. 2025;1(1). 10.64551/001c.147075.

[10] Kalu F, Wolsey C, Enghiad P. Undergraduate nursing students’ perceptions of active learning strategies: a focus group study. Nurse Educ Today. 2023;131:105986. 10.1016/j.nedt.2023.105986. 37857101 10.1016/j.nedt.2023.105986

[11] Pivač S, Skela-Savič B, Jović D, Avdić M, Kalender-Smajlović S. Implementation of active learning methods by nurse educators in undergraduate nursing students’ programs – a group interview. BMC Nurs. 2021;20(1):173. 10.1186/s12912-021-00688-y. 34535119 10.1186/s12912-021-00688-y PMC8449496

[12] Braun V, Clarke V. One size fits all? What counts as quality practice in (reflexive) thematic analysis? One size fits all? What counts as quality practice in (reflexive) thematic analysis? 2021;(Volume 18, Issue 3):328–52. 10.1080/14780887.2020.1769238.

[13] Sandelowski M. Whatever happened to qualitative description? Res Nurs Health. 2000;23(4):334–40. 10.1002/1098-240X(200008)23:4<334::AID-NUR9>3.0.CO;2-G. 10.1002/1098-240x(200008)23:4<334::aid-nur9>3.0.co;2-g 10940958

[14] Kahoot! Learning platform. Available from: https://kahoot.com/. Cited 2026 Feb 18.

[15] Othman SY, Ghallab E, Eltaybani S, Mohamed AM. Effect of using gamification and augmented reality in mechanical ventilation unit of critical care nursing on nurse students’ knowledge, motivation, and self-efficacy: a randomized controlled trial. Nurse Educ Today. 2024;142:106329. 10.1016/j.nedt.2024.106329. 39116661 10.1016/j.nedt.2024.106329

[16] Moreno G, Meneses-Monroy A, Mohamedi-Abdelkader S, Curcio F, Domínguez-Capilla R, Martínez-Rincón C, et al. Virtual active learning to maximize knowledge acquisition in nursing students: a comparative study. Nurs Rep. 2024;14(1):128–39. 10.3390/nursrep14010011. 38251189 10.3390/nursrep14010011 PMC10801574

[17] Al Suliman AS, Al Abdullatif SA, Anna RA, Daniel S, Shahin MAH, Hassan EMG. Enhancing nursing students’ academic performance through the flipped classroom approach: knowledge and perception assessment. Soc Sci Humanit Open. 2025;12:101781. 10.1016/j.ssaho.2025.101781.

[18] Hamarash M, Ibrahim R, Yaas M, Almushhadany OI, Al Mukhtar S. Using peer-assisted learning to enhance clinical reasoning skills in undergraduate nursing students: a study in Iraq. Adv Med Educ Pract. 2025;16:651–62. 10.2147/AMEP.S507996. 40292358 10.2147/AMEP.S507996 PMC12024473

[19] Mohamed SA, Elazim Ibrahim SA, Abdelaalem MM, Mohamed Eldiasty NE. Effect of problem-based learning approach program on meta-cognitive thinking skills and cooperative learning attitude among nursing students: quasi experimental study. BMC Nurs. 2025;24(1):899. 10.1186/s12912-025-03485-z. 40640865 10.1186/s12912-025-03485-z PMC12247313

[20] Bdiri Gabbouj S, Zedini C, Naija W. Effect of simulation-based education of adult BLS-CPR on nursing students’ skills and knowledge acquisition. Adv Med Educ Pract. 2025;16:663–73. 10.2147/AMEP.S500156. 40292357 10.2147/AMEP.S500156 PMC12032964

[21] Alsaraireh A, Madhavanprabhakaran G, Raghavan D, Arulappan J, Khalaf A. Effect of a high-fidelity simulation-based teaching-learning experience (SBTLE) on maternal health nursing students’ knowledge of postpartum hemorrhage, confidence, and satisfaction. Teach Learn Nurs. 2024;19(1):e176–81. 10.1016/j.teln.2023.10.009.

[22] Burgess A, Van Diggele C, Roberts C, Mellis C. Facilitating small group learning in the health professions. BMC Med Educ. 2020;20(S2):457. 10.1186/s12909-020-02282-3. 33272270 10.1186/s12909-020-02282-3 PMC7712521

[23] El-Saftawy E, Latif AAA, ShamsEldeen AM, Alghamdi MA, Mahfoz AM, Aboulhoda BE. Influence of applying VARK learning styles on enhancing teaching skills: application of learning theories. BMC Med Educ. 2024;24(1):1034. 10.1186/s12909-024-05979-x. 39327560 10.1186/s12909-024-05979-x PMC11426201

[24] Velarde-García JF, Álvarez-Embarba B, Moro-Tejedor MN, Rodríguez-Leal L, Arrogante O, Alvarado-Zambrano MG, et al. Barriers and facilitators to the learning and acquisition of research competencies among nursing students through active methodologies: a qualitative study using reflective writing. Healthcare Basel. 2023;11(8):1078. 10.3390/healthcare11081078. 37107912 10.3390/healthcare11081078 PMC10137807

[25] Najjuma JN, Muhumuza A, Santorino D, Sekyere SO, Ocheke AN, Yiltok SJ, et al. Barriers and facilitators to interprofessional simulation-based learning in a Ugandan medical school: a qualitative study. BMC Med Educ. 2024;24(1):1528. 10.1186/s12909-024-06521-9. 39722006 10.1186/s12909-024-06521-9 PMC11669217

[26] Vygotsky LS. Mind in society: the development of higher psychological processes. In: Cole M, John-Steiner V, Scribner S, Souberman E, editors. Cambridge: Harvard University Press; 1978.

[27] Faber TJE, Dankbaar MEW, Van Den Broek WW, Bruinink LJ, Hogeveen M, Van Merriënboer JJG. Effects of adaptive scaffolding on performance, cognitive load and engagement in game-based learning: a randomized controlled trial. BMC Med Educ. 2024;24(1):943. 10.1186/s12909-024-05698-3. 39210381 10.1186/s12909-024-05698-3 PMC11360721

[28] Alsayed S, Alshammari F, Pasay-an E, Dator WL. Investigating the learning approaches of students in nursing education. J Taibah Univ Med Sci. 2021;16(1):43–9. 10.1016/j.jtumed.2020.10.008. 33603631 10.1016/j.jtumed.2020.10.008 PMC7858010

[29] Rodríguez-González R, Martínez-Santos AE, De La Fuente NV, López-Pérez ME, Fernandez-De-La-Iglesia JdelC. Identifying engagement and associated factors in nursing students: an exploratory study. J Prof Nurs. 2023;48:77–83. 10.1016/j.profnurs.2023.06.003. 37775245 10.1016/j.profnurs.2023.06.003

[30] Nagel DA, Penner JL, Halas G, Philip MT, Cooke CA. Exploring experiential learning within interprofessional practice education initiatives for pre-licensure healthcare students: a scoping review. BMC Med Educ. 2024;24(1):139. 10.1186/s12909-024-05114-w. 38350938 10.1186/s12909-024-05114-w PMC10863283

[31] Cenaj D, Schulte-Uentrop L, Kröger LLF, Küllmei J, Haus JM, Moll-Khosrawi P. The effectiveness of Peyton’s 4-step approach to teach resuscitation skills: a randomized controlled clarification study. J Med Educ Curric Dev. 2025;12:23821205251358090. 10.1177/23821205251358090. 40735144 10.1177/23821205251358090 PMC12304607

[32] Maqsood Z, Sajjad M, Yasmin R. Effect of feedback-integrated reflection, on deep learning of undergraduate medical students in a clinical setting. BMC Med Educ. 2025;25(1):66. 10.1186/s12909-025-06648-3. 39810114 10.1186/s12909-025-06648-3 PMC11731358

[33] World Medical Association. World Medical Association Declaration of Helsinki: Ethical principles for medical research involving human subjects. JAMA. 2013;JAMA. 10.1001/jama.2013.281053. 10.1001/jama.2013.281053 24141714

