# Supplementary material for: Exploring the impact of active learning strategies on learning outcomes and educational experiences in undergraduate nursing education: a qualitative descriptive study
Source: BMC Med Educ. 2026 May 23;26:1170. doi: 10.1186/s12909-026-09512-0 (PMC13377703; doi:10.1186/s12909-026-09512-0)
Supplement: Supplementary file 1 — Supplementary Material 1. [file 12909_2026_9512_MOESM1_ESM.zip › AAUP-IRB-R Participant Information Sheet-English Version.docx]

**PARTICIPANT INFORMATION SHEET**

**AAUP-IRB-R Code No.:** ………………………….

**AAUP-IRB-R Date:** ……………………………….

**Study Title:** ***Exploring the Impact of Active Learning Strategies on Learning Outcomes and Educational Experiences in Undergraduate Nursing Education: A Qualitative Case Study***

We would like to invite you to take part in a research study. Before you decide whether to participate, you need to understand why the research is being done and what it would involve. Please take time to read the following information carefully; talk to others about the study if you wish.

Ask us if there is anything that is not clear or if you would like more information. Take time to decide whether or not you wish to take part.

### 1. What is the purpose of this study?

### The purpose of this study is to explore the impact of active learning strategies on the learning outcomes of undergraduate nursing students at the Arab American University, Palestine, from both the students’ and educators’ perspectives. The study seeks to understand how active learning approaches—such as flipped classroom and problem-based learning—affect students’ knowledge, skills, and overall educational experience in nursing education.

### 2. Why is this study important?

### This study is important because it helps to improve the quality of nursing education by identifying how active learning strategies can enhance students’ learning outcomes, critical thinking, clinical competence, and self-directed learning. It also provides valuable insights for educators and institutions on how to shift from traditional teaching methods to more engaging, student-centered approaches that better prepare nursing graduates to deliver safe, effective, and evidence-based patient care.

### 3. What is the procedure that is being tested? (If applicable)

### No medication or medical treatment is being tested in this study. Participation involves taking part in focus group interview lasting approximately 40–60 minutes, during which open-ended questions will be asked about your experiences and perceptions of active learning strategies and their impact on learning outcomes in undergraduate nursing education

### .4. Why have I been invited to participate in this study?

### You have been invited to participate in this study because you are either an undergraduate nursing student or an educator at the Arab American University, Palestine, and your experiences and insights are valuable in understanding how active learning strategies influence nursing education. Your perspective will help identify both the benefits and challenges of applying active learning approaches in the nursing program.

### 5. Who should not participate in the study?

### Individuals who are not undergraduate nursing students or nursing educators at the Arab American University, Palestine, should not participate in this study. Additionally, anyone who does not wish to participate voluntarily or feels uncomfortable discussing their experiences with active learning strategies should not take part.

### 6. Can I refuse to take part in the study?

### Yes. Participation in this study is entirely voluntary. You may refuse to take part or withdraw at any time without any impact on your academic standing, grades, or relationship with the Arab American University, Palestine.

### 7. What will happen to me if I take part?

### If you take part in this study, you will participate in a focus group interview lasting approximately 40–60 minutes. During the interview, you will be asked open-ended questions about your experiences and perceptions of active learning strategies and how they affect learning outcomes in nursing education. Your responses will be confidential, and no personal identifiers will be recorded. The information you provide will be used solely for the purpose of this research.

### 8. How long will I be involved in this study?

### Your participation is limited to the interview session (40–60 minutes). You may be contacted after data analysis to verify that the researchers’ interpretation reflects what you intended (member checking). This is optional, and you may refuse without affecting your main participation.

### 9. What are the possible disadvantages and risks?

### There are minimal risks associated with participating in this study. You may experience mild discomfort or stress when reflecting on your experiences with active learning strategies. There are no physical risks, and all discussions will be kept confidential to protect your privacy. You are free to skip any question or stop the interview at any time if you feel uncomfortable.

### 10. What are the possible benefits to me?

### By participating in this study, you may benefit from the opportunity to reflect on your learning and teaching experiences, gain a deeper understanding of active learning strategies, and contribute to improving nursing education at the Arab American University, Palestine. Your insights may also help shape future teaching practices, benefiting both educators and students in the nursing program.

### 11. Who will have access to my medical records and research data?

### No medical records will be collected in this study.

### Only the research team, specifically the principal investigator, will have access to the research data. All information from interviews will be kept confidential, stored securely on a password-protected computer, and personal identifiers will not be included in any reports or publications.

### 12. Will my records/data be kept confidential?

### Yes. All names and identifying information will be replaced with codes or numbers. Data will be stored on a password-protected device. When reporting results, quotes may be used without any information that could reveal your identity.

### 13. What will happen to any samples I give? (If applicable)

### This study does not involve collecting any samples.

### 14. What will happen if I don’t want to carry on with the study?

### You may withdraw at any time without providing a reason and without affecting your academic standing, grades, or your work or relationship with the Arab American University, Palestine.

### 15. What will happen to the results of the research study?

### The results will be used for academic purposes (e.g., research articles). Results may be published, but no information that identifies you will be disclosed.

### 16. Will I receive compensation for participating in this study?

### No financial compensation will be provided, but your participation is very valuable and will contribute to the development of nursing education.

### 17. Who should I contact if I have additional questions/problems during the study?

### You may contact the researcher:

- Bayan Najdi – Phone: +972 523407739

1. **Who should I contact if I am unhappy with how the study is being conducted?**

Institutional Review Board – Ramallah

Arab American University

Email: [IRB-R@aaup.edu](mailto:IRB-R@aaup.edu)
