# Supplementary material for: Exploring the impact of active learning strategies on learning outcomes and educational experiences in undergraduate nursing education: a qualitative descriptive study
Source: BMC Med Educ. 2026 May 23;26:1170. doi: 10.1186/s12909-026-09512-0 (PMC13377703; doi:10.1186/s12909-026-09512-0)
Supplement: Supplementary file 1 — Supplementary Material 1. [file 12909_2026_9512_MOESM1_ESM.zip › COREQ reporting guidelines.docx]

**Table 1**

Consolidated criteria for reporting qualitative studies (COREQ): 32-item checklist

| No | Item | Guide questions/description |
| --- | --- | --- |
| Domain 1: Research team and reflexivity |  |  |
| Personal Characteristics |  |  |
| 1 | Interviewer/facilitator | Methodology – Data collection (p. 5) |
| 2 | Credentials | Title page |
| 3 | Occupation | Not reported |
| 4 | Gender | Not reported |
| 5 | Experience/training | Methods – Data collection (p. 5) |
| Relationship with participants |  |  |
| 6. | Relationship established | Not established prior |
| 7. | Participant knowledge of the interviewer | Methodology – consent (p. 5) |
| 8. | Interviewer characteristics | Methodology – Trustworthiness (p. 6) |
| Domain 2: study design |  |  |
| Theoretical framework |  |  |
| 9. | Methodological orientation and Theory | Introduction_ (p2) Methodology – (p. 4,7) |
| Participant selection |  |  |
| 10. | Sampling | Methodology – (p. 4) |
| 11. | Method of approach | Methodology – Recruitment procedure (p. 4,5) |
| 12. | Sample size | Methodology – (p. 4) |
| 13. | Non-participation | None |
| Setting |  |  |
| 14. | Setting of data collection | Methodology – (p. 5) |
| 15. | Presence of non-participants | No one else present besides the participants and researchers Methodology – (p. 5) |
| 16. | Description of sample | (p. 7,8) – Table 1 (p. 7) |
| Data collection |  |  |
| 17. | Interview guide | \|  \| \| --- \|  \| Methodology – (p. 4) \| \| --- \| |
| 18. | Repeat interviews | No repeat interviews carried out |
| 19. | Audio/visual recording | Methodology – (p. 5,6) |
| 20. | Field notes | Methodology – (p. 5,6) |
| 21. | Duration | Methodology – (p. 5,6) |
| 22. | Data saturation | Methodology – (p. 5) |
| 23. | Transcripts returned | \|  \| \| --- \|  \| Member checking (p. 6,7) \| \| --- \| |
| Domain 3: analysis and findings |  |  |
| Data analysis |  |  |
| 24. | Number of data coders | Methodology – (p.6) |
| 25. | Description of the coding tree | \|  \| \| --- \|  \| Results – Figures 1–4 (p. 9,11,15,22) \| \| --- \| |
| 26. | Derivation of themes | \|  \| \| --- \|  \| Methodology – Thematic analysis (p. 6-8) \| \| --- \| |
| 27. | Software | Methodology – NVivo (p. 6) |
| 28. | Participant checking | \|  \| \| --- \|  \| Member checking (p. 6,7) \| \| --- \| \|  \| \| |
| Reporting |  |  |
| 29. | Quotations presented | Results – Themes sections (p. 9–25) |
| 30. | Data and findings consistent | Results & Discussion (p. 9–32) |
| 31. | Clarity of major themes | Results – Themes 1–5(p. 9–25) |
| 32. | Clarity of minor themes | \|  \| \| --- \|  \| Results – Subthemes (p. 9–25) \| \| --- \| |
