# Supplementary material for: Exploring the impact of active learning strategies on learning outcomes and educational experiences in undergraduate nursing education: a qualitative descriptive study
Source: BMC Med Educ. 2026 May 23;26:1170. doi: 10.1186/s12909-026-09512-0 (PMC13377703; doi:10.1186/s12909-026-09512-0)
Supplement: Supplementary file 1 — Supplementary Material 1. [file 12909_2026_9512_MOESM1_ESM.zip › Focus Group Interview Guide 1 Undergraduate Nursing Students Appendix A.docx]

**Study title:** The Impact of Active Learning Strategies on Learning Outcomes of Undergraduate Nursing Students: A Case Study at Arab American University – Palestine **Focus Group Interview Guide 1**

**Undergraduate Nursing Students’ Experiences with Active Learning**

**Adapted with modifications from:** Pivač et al. (2021)
**Estimated duration:** 45–60 minutes
**Group size:** 6–8 students

**Introduction (5 minutes)**

“Welcome, and thank you for joining this discussion. We’re here to explore how *active learning* activities like problem-based learning, simulations, group work, or flipped classrooms affects your learning and development as nursing students at the Arab American University of Palestine. There are no right or wrong answers; we just want to understand your experiences and opinions.”

- Confidentiality and consent for audio recording (I will explain)
- Ask for each participant’s name, study year, and one sentence describing their learning experience this semester.

**Warm-up / Icebreaker**

**Prompt:** How would you describe “active learning” in your own word?
(If a participant mentions an active learning activity, use that to transition into the next section.)

**Core Questions (35 minutes)**

1. **Familiarity and Recognition**
   - How familiar are you with *active learning strategies*?
   - Can you describe examples of activities that you think used active learning?
     What made you feel that this activity was “active”?
   1. How did you participate during this activity?
   2. Can you walk me through what you were asked to do in that session?
   3. What skills or thinking did the activity require from you?
   4. How was this activity different from regular lectures?
   5. How did your classmates interact during this activity?
   6. How did the instructor guide or facilitate the activity?
2. **Perceived Impact**
   - Think about one memorable learning experience—either the best or the worst—you’ve had as a nursing student. What made it stand out for you?
   - Compared with lecture-based teaching, how have active learning strategies affected your understanding or retention of information?
   - How have they influenced your critical thinking, problem-solving, or confidence in clinical settings?
3. **Helpful vs. Less Helpful Activities**
   - Which specific activities or methods have been most helpful to your learning?
   - Which ones were least helpful or difficult, and why?
4. **Structure and Delivery**
   - What balance between lectures and active sessions works best for you?
     When do you feel lectures are most helpful for your learning?
   1. In what situations do active learning activities work better for you?
   2. Can you describe an ideal class session for you — how much time would be lecture vs. activity?
   3. Do you prefer shorter lectures with activities in between, or longer lectures with activities at the end? Why?
   4. How does the balance affect your focus or understanding?
   5. Have you experienced a course where the balance felt “just right”? What was it like?
   - How do you feel about being asked to prepare readings or materials before class?
5. **Learning Environment**
   - What classroom conditions help you learn best during active learning sessions?
   - (Prompts: seating arrangement, space, temperature, group size, instructor’s role.)
6. **Engagement and Motivation**
   - What motivates you most to participate in active learning sessions?
   - How do active learning sessions make you feel (for example: encouraged, motivated, confidence and readiness for clinical practice, confused, or discouraged)?
     Can you explain what factors contribute to these feelings?”
7. What parts of the session made you feel this way?
8. Can you give an example of a moment you felt particularly motivated/confused?
9. How did the instructor’s approach affect your feelings?
10. How did group work or class interaction influence your experience?
11. **Final Reflections**
    - Overall, what changes would make active learning more effective in your nursing courses?
12. Can you give an example to clarify your point?
13. What experiences made you feel this way?
14. How does this influence your learning?
15. What would you prefer instead?
    - Discuss how active learning experiences in your courses have impacted your preparedness for clinical practice. Can you give examples of situations where it helped or didn’t help?
    1. Can you describe a specific activity that made you feel more prepared?
    2. Were there any moments where active learning didn’t help you feel ready? Why?
    3. How does your experience in active learning translate into practical skills or confidence in clinical settings?

**Closing (5 minutes)**

- Summarize key ideas shared.
- Invite any last comments.
- Thank participants for their valuable input.

**Reference:**
*Adapted from Pivač et al. (2021). Modified for the Palestinian nursing education context at Arab American University.*

Pivač, S., Skela-Savič, B., Jović, D. *et al.* Implementation of active learning methods by nurse educators in undergraduate nursing students’ programs – a group interview. *BMC Nurs* **20**, 173 (2021). https://doi.org/10.1186/s12912-021-00688-y
