# Supplementary material for: Exploring the impact of active learning strategies on learning outcomes and educational experiences in undergraduate nursing education: a qualitative descriptive study
Source: BMC Med Educ. 2026 May 23;26:1170. doi: 10.1186/s12909-026-09512-0 (PMC13377703; doi:10.1186/s12909-026-09512-0)
Supplement: Supplementary file 1 — Supplementary Material 1. [file 12909_2026_9512_MOESM1_ESM.zip › Focus Group Interview Guide 2 educators Appendix B.docx]

**Study title:** The Impact of Active Learning Strategies on Learning Outcomes of Undergraduate Nursing Students: A Case Study at Arab American University – Palestine **Focus Group Interview Guide 2**

**Nurse Educators’ Perspectives on Implementing Active Learning**

**Adapted with modifications from:** **Pivač et al. (2021), Kalu et al. (2023)
Estimated duration: 45–60 minutes
Group size: 4–6 educators**

**Introduction (5 minutes)**

“This discussion focuses on your experiences as educators implementing active learning strategies in nursing courses. I’d like to understand how these methods influence students’ learning outcomes, as well as what supports or challenges you encounter.”

(I have to Explain) confidentiality, voluntary participation, and recording.

**Core Questions (40 minutes)**

1. Definition and Practice
   - How do you personally define *active learning* in nursing education?
   - Which strategies (e.g., simulation, case study, flipped classroom, peer learning) do you use most often? Why?
   - How you align them with your teaching practice and assessment?
2. Assessing students

- What methods of observation do you typically use for assessing students during active learning sessions, both formative and summative?

1. For example, do you use direct observation, student self-reflection, peer evaluation, quizzes, or other methods?”
2. How often do you provide feedback during these sessions?”
3. Observed Student Outcomes
   - What changes have you noticed in students’ engagement, understanding, or performance when using active learning strategies?
   - How do these strategies affect students’ clinical reasoning and decision-making?
4. Can you give examples of activities that led to increased engagement or understanding?”
5. How do students respond differently compared to traditional lectures?”
6. Have these strategies influenced students’ confidence or skills in clinical practice?”
7. Facilitators and Barriers
   - What factors make it easier for you to integrate active learning into your teaching?
   1. Can you describe situations where it felt easier to use active learning?
   2. What conditions or circumstances helped you implement it?
   3. How did your preparation or experience affect your ability to use active learning?
   4. Were there aspects of the students’ participation that made it easier?
   - What obstacles or institutional constraints do you face (e.g., time, resources, class size, training)?
8. Instructional Environment
   - What classroom or institutional conditions best support active learning?
   1. Can you describe a situation or setting where active learning worked well?
   2. What aspects of the classroom or institution helped facilitate active learning?
   3. How did timing, scheduling, or course organization affect your ability to use active learning?
   4. Were there features of the learning environment that made activities easier or more effective?
   5. Is there anything else about the setting or context that supported your teaching?
   - How do you adapt when the environment is less ideal?
9. Evaluation and Reflection
   - How do you evaluate whether your active learning sessions were successful?
   - How do you adjust your teaching based on students’ feedback or performance?
10. Professional Development and Support
    - What kind of support or training would help you improve the implementation of active learning?
    - What recommendations would you make to strengthen active learning in the nursing curriculum?

**Closing (5 minutes)**

- Is there anything else you’d like to add about your experience using active learning in nursing education?

**Reference:**

*Adapted from Pivač et al. (2021) and Kalu et al. (2023); modified for the Palestinian nursing education context at Arab American University.*

Pivač, S., Skela-Savič, B., Jović, D. *et al.* Implementation of active learning methods by nurse educators in undergraduate nursing students’ programs – a group interview. *BMC Nurs* **20**, 173 (2021). https://doi.org/10.1186/s12912-021-00688-y

*Kalu, F., Wolsey, C., & Enghiad, P. (2023). Undergraduate nursing students’ perceptions of active learning strategies: A focus group study. Nurse Education Today, 131, 105986. https://doi.org/10.1016/j.nedt.2023.105986*
