# Supplementary material for: Exploring the impact of active learning strategies on learning outcomes and educational experiences in undergraduate nursing education: a qualitative descriptive study
Source: BMC Med Educ. 2026 May 23;26:1170. doi: 10.1186/s12909-026-09512-0 (PMC13377703; doi:10.1186/s12909-026-09512-0)
Supplement: Supplementary file 1 — Supplementary Material 1. [file 12909_2026_9512_MOESM1_ESM.zip › R-2025 A 97 N (2).pdf]

IRB Approval Letter

Study Title: "The Impact of Active Learning Strategies on Learning Outcomes of Undergraduate Nursing Students: A Case Study at Arab American University – Palestine".

Submitted by: **Bayan Mohammad Najdi**

Date received: 18th November 2025

Date reviewed: 26th November 2025

Date approved: 1st December 2025

Your Study titled **"The Impact of Active Learning Strategies on Learning Outcomes of Undergraduate Nursing Students: A Case Study at Arab American University – Palestine"** with the code number **"R-2025/A/97/N"** was reviewed by the Arab American University Institutional Review Board - Ramallah and it was approved on the 1st of December 2025.

Sajed Ghawadra, PhD
IRB-R Chairman
Arab American University of Palestine

[illegible]

الجامعة العربية الأمريكية - فلسطين
مجلس اخلاقيات البحث العلمي - رام الله
IRB-R
ARAB AMERICAN UNIVERSITY-PALESTINE
INSTITUTIONAL REVIEW BOARD - RAMALLAH

General Conditions:

1. Valid for 6 months from the date of approval.
2. It is important to inform the IRB-R with any modification of the approved study protocol.
3. The Bord appreciates a copy of the research when accomplished.
